# A case of congenital *genu recurvatum* in a newborn

**DOI:** 10.11604/pamj.2022.43.179.36519

**Published:** 2022-12-06

**Authors:** Umadevi Ramachandran, Krishna Prasanth Baalann

**Affiliations:** 1 Department of Community Medicine, Sree Balaji Medical College and Hospital, Biher, Chennai, Tamilnadu, India

**Keywords:** Congenital *genu recurvatum*, hyperextension, poliomyelitis

## Image in medicine

*Genu recurvatum* is a highly complex knee-joint deformity. The knee bends backward with an extensive extension of the tibiofemoral joint in this condition. It is most common in women. Congenital *genu recurvatum* is a rare condition that affects about one in every 100,000 births. It can be hereditary or acquired. The most common cause of acquired *genu recurvatum* is poliomyelitis. Other causes include epiphyseal growth defects, malunited fractures, inherent ligament laxity, injury, misalignment of the ankle joint, joint instability, anterior cruciate ligament tear, weakness in the hip extensor muscles or quadriceps femoris muscle, connective tissue diseases, and a difference in lower limb length. Pain, weakness, instability, an extension gait pattern, a leg-length discrepancy, pining in front of the knee, and a decreased range of motion are the most common symptoms of this condition. It is diagnosed by evaluating the increase in patient's heel height, to measure the extent of hyperextension of the knee. At 39 weeks of pregnancy, the neonate was delivered via normal vaginal delivery. The deformity was noted. Manipulation and serial correction with Plaster of Paris application were used in treatment, which was changed every three weeks until the desired outcome was archived. Patients were followed up on until they were able to walk. To correct the deformities completely, an average of six casts were required over the course of 17 weeks. *Genu recurvatum* being congenital deformity it imposes a threat to the future generations. As polio being a major preventable risk factor, the social awareness regarding this correctable deformity is a need.

**Figure 1:**
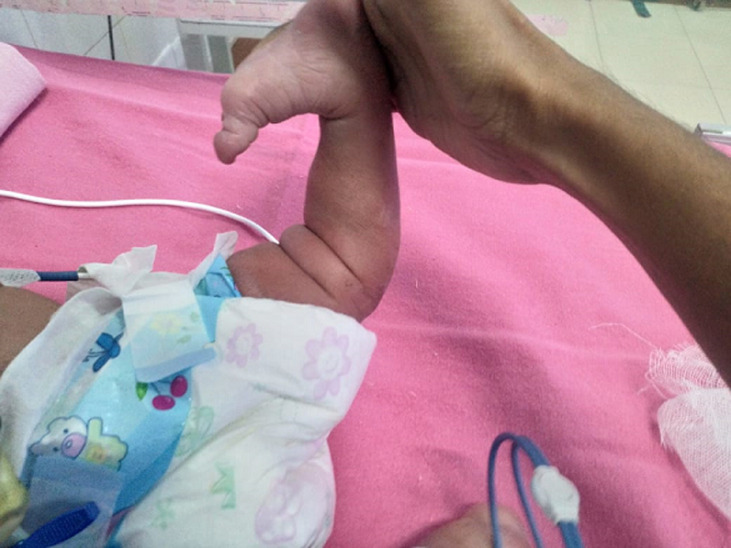
hyperextension of knee joint-*genu recurvatum*

